# Pharmacists’ perspectives on potential pharmacist prescribing: a nationwide survey in the Netherlands

**DOI:** 10.1007/s11096-024-01842-7

**Published:** 2024-12-01

**Authors:** Bilge Kaymakci, Daphne Philbert, Ankie C. M. Hazen, Mette Heringa, Henk-Frans Kwint, Dorien L. M. Zwart, Liset van Dijk, Sofia Kälvemark Sporrong, Thomas G. H. Kempen

**Affiliations:** 1 Division of Pharmacoepidemiology and Clinical Pharmacology, Utrecht Institute for Pharmaceutical Sciences, Utrecht University, Utrecht, The Netherlands; 2 Julius Center for Health Sciences and Primary Care, University Medical Center Utrecht, Utrecht, The Netherlands; 3 SIR Institute for Pharmacy Practice and Policy, Leiden, The Netherlands; 4 Nivel, Netherlands Institute for Health Services Research, Utrecht, The Netherlands; 5 Groningen Research Institute of Pharmacy, Unit of PharmacoTherapy, Epidemiology and Economics, University of Groningen, Groningen, The Netherlands; 6 Department of Pharmacy, Uppsala University, Uppsala, Sweden

**Keywords:** Drug prescribing, Health policy, Non-medical prescribing, Pharmacists, Surveys and questionnaires, Task shifting

## Abstract

**Background:**

Pharmacist prescribing legislation aims to enhance healthcare quality and accessibility. However, in many countries, like the Netherlands, it has not yet been legally established.

**Aim:**

To investigate pharmacists’ perspectives on potential pharmacist prescribing in the Netherlands.

**Method:**

An online survey using a questionnaire that was distributed via e-mail and electronic newsletters to most practicing pharmacists in the Netherlands during October and November 2023. The questionnaire was based on previous literature, further developed during an international conference with pharmacists and piloted with Dutch pharmacists. Agreement with statements about potential prescribing models, settings, preconditions, and perceived benefits and risks was measured using a 4-point Likert scale. Data were analysed descriptively.

**Results:**

In total, 625 participants from community pharmacy (n = 432; 69.1%), hospital pharmacy (n = 149; 23.8%), or other/combined settings (n = 44; 7.0%) completed the questionnaire. Most pharmacists (somewhat) agreed with the introduction of an independent prescribing model with limitations (n = 538; 86.1%) or a model dependent on collaborative agreements with physicians (n = 471; 75.4%). A minority (n = 245; 39.2%) supported independent prescribing with diagnostic authority. The precondition that participants most frequently (somewhat) agreed with was access to health records (n = 607; 97.1%). The most (somewhat) agreed-upon benefits were enhanced professional position of pharmacists (n = 574; 91.8%) and reduced workload for other prescribers (n = 573; 91.7%). Increased workload for pharmacists (n = 495; 79.2%) was the most (somewhat) agreed-upon identified risk.

**Conclusion:**

Pharmacists in the Netherlands are generally supportive of an independent but limited or collaborative pharmacist prescribing model. These findings support further investigations into the potential introduction of pharmacist prescribing legislation.

**Supplementary Information:**

The online version contains supplementary material available at 10.1007/s11096-024-01842-7.

## Impact statements

- The strong support by pharmacists for pharmacist prescribing highlights the potential to influence stakeholders, paving the way for discussions on implementing prescribing legislation.
- Many pharmacists perceive that prescribing privileges could potentially decrease the workload of other healthcare professionals, improve patient access to care, and potentially enhance overall quality of care.
- Policymakers should carefully evaluate key preconditions, such as access to health records, while considering potential risks, such as increased pharmacist workload and conflicts of interest, to ensure that the introduction of pharmacist prescribing contributes positively to clinical outcomes.

## Introduction

The aging global population and increasing healthcare needs, combined with a shortage of healthcare professionals, present significant challenges for national health systems [1–5]. Optimizing healthcare professional expertise can help to ensure healthcare quality and accessibility. Non-medical prescribing—by professionals other than medical doctors—is one approach to use healthcare professionals’ skills more effectively, improving access to timely care, and reducing physician workload [6, 7]. Some countries have granted pharmacists the right to prescribe medication with models and policies varying across countries [8–10]. In the United Kingdom (UK), independent prescribing allows pharmacists to manage any clinical condition and prescribe any medication within their clinical competence [6]. In contrast, other countries restrict prescribing to specific formularies, listing the medications or conditions for which prescriptions can be made [9, 10]. Another model is collaborative or dependent prescribing, which involves prescribing in the context of protocols, agreements or collaboration with physicians or multidisciplinary teams, e.g., the current pharmacist prescribing model in New Zealand [7, 8]. In Canada and the United States of America (USA), pharmacist prescribing rights vary significantly between regions [9, 10].

A Cochrane review of 20 clinical trials on pharmacists prescribing concluded that it is as effective as physician prescribing in terms of cardiovascular risk factors, medication adherence, patient satisfaction, and health-related quality of life [11]. Prescribing by pharmacists also seems beneficial in improving access to medication [12]. Findings from a recent Canadian trial indicate that prescribing by community pharmacists reduces the risk of stroke in patients with atrial fibrillation [13]. In another recent trial from the UK, pharmacists’ prescribing in nursing homes was safe and welcomed by care home staff and general practitioners [14]. Pharmacist prescribing reduced the Drug Burden Index, a measure of anticholinergic and sedative drug exposure, but it did not reduce falls in care home residents over the six month follow-up.

However, the implementation of pharmacist prescribing faces barriers, including limited access by pharmacists to health records, workforce shortages, inadequate funding, lack of physician support, and legal concerns like accountability [8, 15, 16]. Opinions about pharmacist prescribing vary, primarily studied in countries that had introduced or were about to introduce such legislation. Some stakeholders express concerns about pharmacists’ clinical competence and potential conflicts of interest, while others acknowledge pharmacists’ expertise and value the increased patient access to care [8, 15–17]. Pharmacists often express willingness to obtain prescribing rights, because of the potential to improve patient outcomes and reduce other healthcare professionals’ workload [8, 15, 16]. Less is known about what pharmacists in countries without prescribing legislation think about potential pharmacist prescribing. In the Netherlands, pharmacist prescribing has not been legally established, even though pharmacists are increasingly involved in pharmacotherapeutic decisions through collaboration with physicians [18]. Examples include pharmacotherapy audit meetings between community pharmacists and general practitioners, medication reconciliation in hospital, and patient-facing clinics managed by general practice-based pharmacists [19–21]. In these contexts, pharmacists currently engage in informal prescribing practices where they write prescriptions that are then authorized by a physician before dispensing [22]. Understanding the pharmacists’ perspectives is crucial for informing policymakers about the potential to introduce prescribing models [8, 23]. This may help shape future policies and practices regarding pharmacists prescribing.

### Aim

To investigate pharmacists’ perspectives on potential pharmacist prescribing in the Netherlands.

### Ethics approval

Approval for this study was granted on 18 October 2023 (UPF2311) by the Institutional Review Board, Division of Pharmacoepidemiology & Clinical Pharmacology, Department of Pharmaceutical Sciences, Utrecht University. Informed consent was obtained from all participants.

## Method

### Study design, setting and population

An online nationwide cross-sectional survey study was conducted and reported following the Consensus-Based Checklist for Reporting of Survey Studies (CROSS) [24]. All approximately 4000 practicing pharmacists in the Netherlands [25–27] were eligible for this study. Practicing pharmacists were defined as licensed pharmacists who are involved in the provision of pharmaceutical care for individual patients, in any healthcare setting: community pharmacy, outpatient and inpatient hospital pharmacy, or other healthcare settings (e.g., nursing home or general practice).

### Questionnaire development

**Fig. 1:**
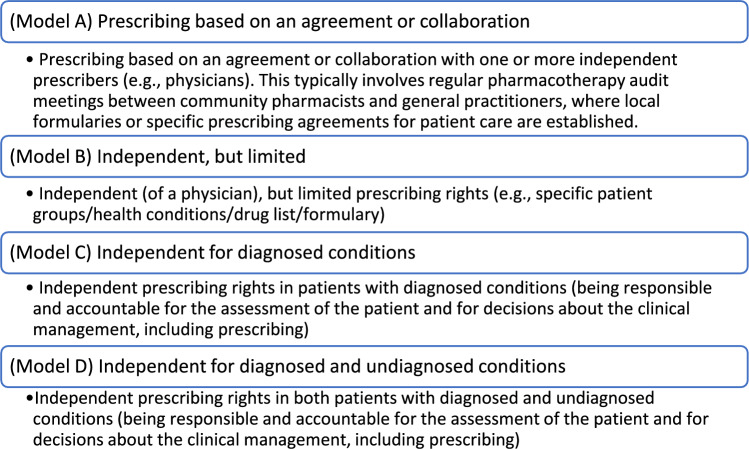
Potential models of pharmacist prescribing rights in the Netherlands with varying difference of independence, as presented in the questionnaire

The Dutch questionnaire was adapted from an English version that had previously been developed. First, a Swedish version was developed at Uppsala University, based on previous literature [8, 16, 17], and piloted among clinical pharmacists in Sweden in 2022 [28]. During a 12-h workshop at the Pharmaceutical Care Network Europe (PCNE) conference in Hillerød, Denmark (8–11 February 2023), the questionnaire was translated into English and adapted. The workshop, moderated by two researchers (TK and SKS), involved seventeen pharmacists and academics from eleven countries (across Europe and Taiwan) who collaborated to refine the English version for use in countries where pharmacists have no or limited prescribing rights. This questionnaire addressed participant demographics, pharmacist prescribing models, settings, preconditions for introduction, and associated benefits and risks. It featured four pharmacist prescribing models inspired by practices in New Zealand (model A), Ontario, Canada (model B), and the UK (models C-D; Fig. 1).

The questionnaire included 4-point Likert scale questions, ranging from "Disagree" to "Agree," with the additional option of "Don't know/No opinion," and an open text section for comments. The Dutch version was translated from the English using DeepL Translator [29]. Errors were corrected, some questions were adjusted for Dutch language nuances, and country-specific demographic questions were added. The survey was reviewed and revised by three researchers (BK, TK, DP) and then sent to other team members (HFK, AH, MH, LvD, DZ) for further input. Their feedback was incorporated, resulting in the final version of fifteen questions (Supplementary Material, Appendix 1).

### Piloting questionnaire

The Dutch survey was piloted in October 2023 with three pharmacists from inpatient and outpatient hospital pharmacy settings. This pilot tested for the comprehensibility of the questionnaire content, including the introduction and questions, as well as the survey's formatting and functionality. Changes to participant demographic questions and minor textual adjustments were made; examples of pharmacist prescribing models from New Zealand (model A), Ontario, Canada (model B) and the UK (model C-D) were added before the model-related questions.

### Participant recruitment and data collection

Practicing pharmacists were recruited through the Utrecht Pharmacy Practice network for Education and Research (UPPER), the Royal Dutch Pharmacists Association (KNMP), the Dutch Association of Hospital Pharmacists (NVZA), and an informal network of general practice-based pharmacists, ensuring a broad reach across the target group. The UPPER network [30] has an e-mail database of over 2000 members, the KNMP/NVZA network comprises 5470 members, and the network of general practice-based pharmacists includes fifteen members. An e-mail was sent to all UPPER network members and general practice-based pharmacists, inviting them to participate in the study. KNMP and NVZA added a survey link to their newsletter. There is overlap between the UPPER and KNMP/NVZA networks, leading to some pharmacists receiving multiple participation requests. By utilizing these four networks of pharmacists, the aim was to maximize exposure to the survey and increase the likelihood of participation. In all four approaches, potential participants received study information and a link to Qualtrics (Provo, Utah, USA), an online survey tool used for the questionnaire. By submitting the completed questionnaire, participants consented to participate and understood that due to data anonymity, their responses could not be withdrawn once submitted. Detailed participant information was provided in Qualtrics to ensure informed consent before participation. The survey was open for four weeks in October—November 2023. A follow-up email and newsletter were sent two weeks after the initial questionnaire opening. Pharmacists were explicitly asked to complete the questionnaire only once.

### Data-analysis

Completed questionnaires were collected in Qualtrics and exported to IBM Statistical Package for Social Sciences (SPSS) version 29 (Armonk, New York, USA) for analysis. Participants were excluded if they were not practicing pharmacists. Descriptive statistics were applied to all quantitative results. The open-text comments were analysed, categorized and presented descriptively where applicable or textually summarized. Pre-defined subgroup analyses were conducted by practice setting, frequency of direct patient contact and years of working experience to explore how these characteristics were associated with agreement with different prescribing models. For these subgroup analyses, the 4-point Likert scale was simplified by combining 'Somewhat disagree' with 'Disagree' and 'Somewhat agree' with 'Agree'. Differences were assessed using chi-square tests (statistical difference at *p* < 0.05), with post hoc analyses to adjust for multiple comparisons.

## Results

### Participants

At least 2000 pharmacists were directly approached by e-mail, with additional indirect recruitment through electronic newsletters. Out of 741 questionnaires that were started, 625 were completed and included. These 625 participants correspond to approximately 15% of the estimated 4000 practicing pharmacists in the Netherlands. Of these participants, 59.2% (n = 370) were female, 44.3% (n = 280) were aged 40 or younger, and the majority (70.2%, n = 439) worked in community pharmacy (Table 1). Of all participants, 67.7% (n = 423) had over ten years of practice experience.

**Table 1:** Demographic characteristics of survey participants (n = 625)

Characteristic		n (percentage)
Gender	Male	25 (40.3%)
	Female	370 (59.2%)
	Non-binary	1 (0.2%)
	Do not want to answer	2 (0.3%)
Age group (years)	24–30	83 (13.3%)
	31–40	194 (31.0%)
	41–50	189 (30.2%)
	> 50	159 (25.4%)
Years of working experience	0–3	107 (17.2%)
	4–9	95 (15.2%)
	> 10	423 (67.7%)
Working setting	Community pharmacy	432 (69.1%)
	Outpatient hospital pharmacy	24 (3.8%)
	Inpatient hospital pharmacy	125 (20.0%)
	Other^a^	44 (7.0%)
Highest finished degree	Master of Pharmacy	110 (17.6%)
	Post-graduate community pharmacy degree and/or registration	367 (58.7%)
	Additional community pharmacy specialisation	7 (1.1%)
	Post-graduate inpatient hospital pharmacy degree and/or registration	113 (18.1%)
	Clinical pharmacology specialisation	28 (4.5%)

^a^Institution (e.g., nursing home), general practice and combined settings/workplaces

### Different models and settings of pharmacist prescribing

**Fig. 2:**
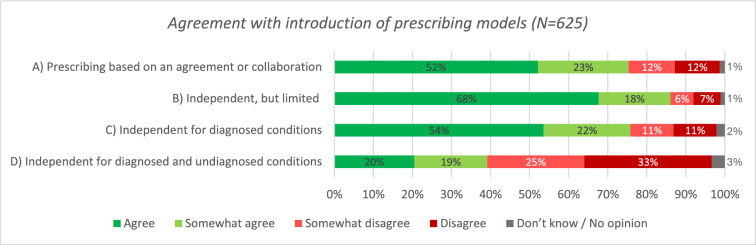
Participant’s level of agreement with the introduction of the four presented potential models of pharmacist prescribing (n = 625)

Of the 625 participants, 538 (86.1%) agreed or somewhat agreed with the implementation of independent but limited prescribing in the Netherlands (model B; Fig. 2). Many participants (n = 474; 75.8%) agreed or somewhat agreed with independent prescribing for patients with diagnosed conditions (model C). A similar number, 471 participants (75.4%), expressed to (somewhat) agree with prescribing rights based on collaborative agreements (model A). Finally, 245 participants (39.2%) agreed or somewhat agreed with the implementation of independent prescribing for both diagnosed and undiagnosed conditions (model D). Nearly all participants (n = 616; 98.6%) agreed or somewhat agreed with at least one proposed prescribing model. Few participants (n = 9; 1.4%) disagreed with all proposed models and highlighted (through open-text comments) the importance of separation of tasks and responsibilities between physicians and pharmacists and the potential conflict of interest as key concerns.

Community pharmacists showed higher agreement with the introduction of the three independent pharmacist prescribing models than inpatient hospital pharmacists (93.5% vs. 73.1%, 85.6% vs. 47.1%, and 54.0% vs. 19.3%, *p* < 0.001, for model B, C and D respectively, Supplementary Material, Appendix 2, Table S2). Among the participants, 89.9% (n = 562) supported implementing prescribing rights within community pharmacy, 80.6% (n = 504) in primary care, 77.6% (n = 485) in nursing homes, and 72.0% (n = 450) in secondary care. Additionally, pharmacists with daily direct patient contact agreed to a higher degree with the introduction of pharmacist prescribing than those who (almost) never had direct patient contact (90.3% vs. 67.0%, 84.1% vs. 51.1%, and 47.5% vs. 18.1%, *p* < 0.001, for model B, C and D respectively, Table S4). There were no differences between subgroups based on years of working experience (Table S6).

### Pharmacist agreement with performing tasks related to pharmacist prescribing

**Fig. 3:**
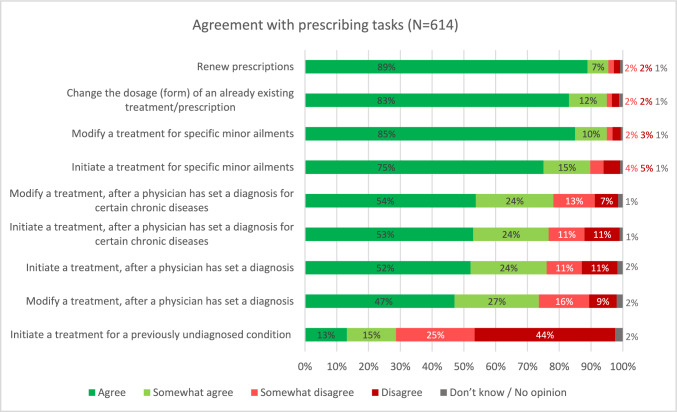
Participants’ level of agreement with tasks regarding pharmacist prescribing in order of highest agreement (n = 614; nine participants who disagreed with all proposed models in the previous question automatically skipped this question)

Most pharmacists agreed or somewhat agreed with carrying out prescription renewals (n = 586; 93.8%) and changing the dosage of an already existing treatment or prescription (n = 583; 93.3%; Fig. 3). Many participants indicated that they were already performing these tasks in practice, although without having formal prescribing rights. Furthermore, a majority agreed with pharmacist involvement in modifying or initiating treatments for chronic conditions diagnosed by physicians (76.8%, n = 480 to 74, 7%, n = 467). There was little agreement with initiating treatment for undiagnosed conditions (n = 176; 28.2%).

### Pharmacist agreement with required preconditions regarding the introduction of pharmacist prescribing

**Fig. 4:**
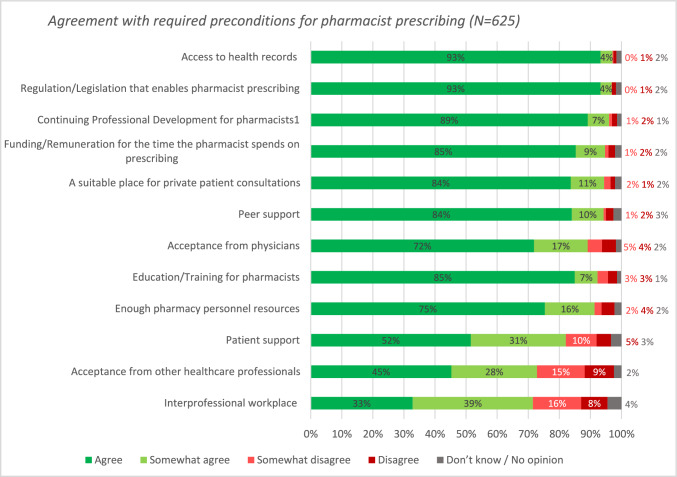
Participants’ level of agreement with required preconditions regarding the introduction of pharmacist prescribing in order of highest agreement (n = 625). ^1^A formal system or framework that involves the tracking and documenting of skills, knowledge and experience gained, beyond any initial education and training

Most of the participants agreed or somewhat agreed with the pre-listed conditions considered important for the introduction of pharmacist prescribing (Fig. 4), with highest agreement for access to health records (n = 607; 97.1%;) and regulation/legislation enabling pharmacist prescribing (n = 625; 96.8%). Many participants (n = 589; 94.2%) agreed with the importance of gaining acceptance from physicians. However, it was emphasized in free text that pharmacists should not wait for full acceptance before implementing prescribing.

### Pharmacist agreement with potential benefits and risks of pharmacist prescribing

**Fig. 5:**
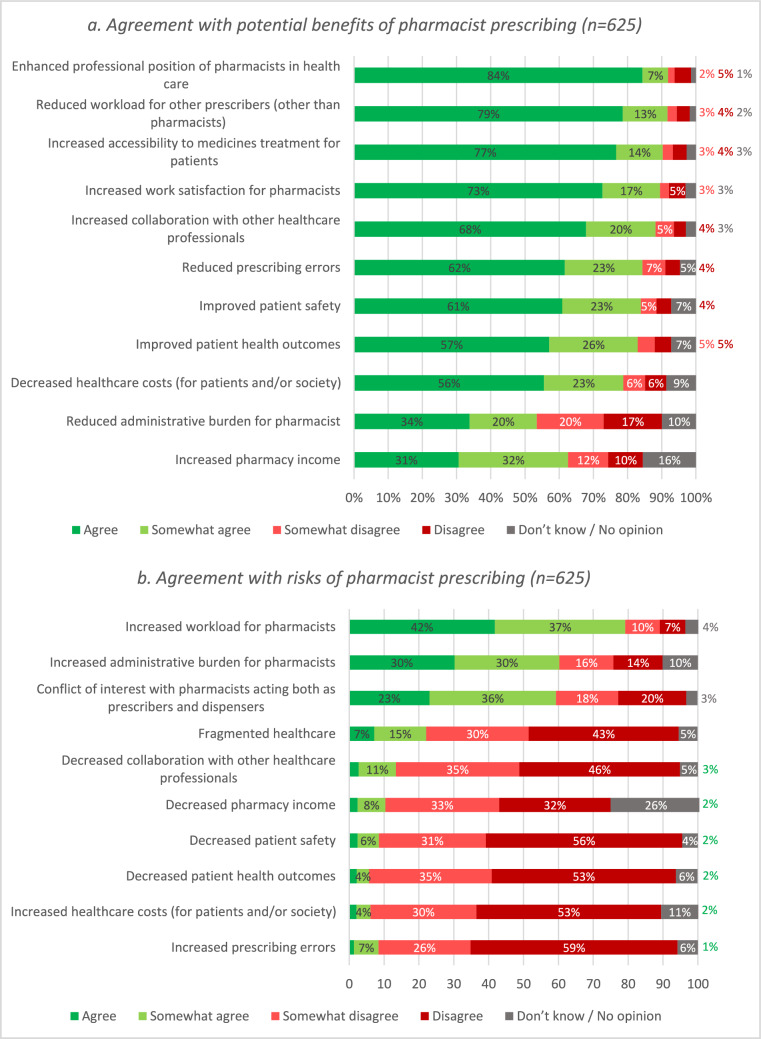
Participants’ level of agreement with potential benefits (**a**) and risks (**b**) of introducing pharmacist prescribing in order of highest agreement (n = 625)

The surveyed pharmacists (somewhat) agreed with multiple pre-listed benefits of pharmacist prescribing (Fig. 5a). These included enhanced professional position of pharmacists in healthcare (n = 574; 91.8%), and reduced workload for prescribers other than pharmacists (n = 573; 91.7%). There was also support for increased accessibility to medical treatment for patients (n = 564; 90.2%) and work satisfaction for pharmacists as a potential benefit.

Participants’ agreement with pre-listed risks associated with pharmacist prescribing highlighted that they identified increased workload for pharmacists (n = 495; 79.2%) and an elevated administrative burden (n = 377; 60.3%) as primary concerns (Fig. 5b). Participants agreed or somewhat agreed with the risk of potential conflict of interest when pharmacists assume dual roles as prescribers and dispensers (n = 371; 59.4%). A small percentage of the participants (n = 36; 5.8%) expressed concerns about potential declines in patient health outcomes due to pharmacist prescribing.

## Discussion

### Statement of key findings

This survey study investigated the pharmacists’ perspectives on potential pharmacist prescribing in the Netherlands. Most participants seemed to support introducing prescribing in an independent but limited or collaborative model. There was more support for the introduction of pharmacist prescribing among pharmacists with daily direct patient contact compared to those with (almost) no direct patient contact. All settings seemed suitable to introduce pharmacist prescribing. The precondition that pharmacists most often indicated as important was access to health records. An improved professional position for pharmacists and increased accessibility to medication treatment for patients were the most prevalent potential benefits. A potential increase in pharmacist workload emerged as the most anticipated risk.

### Strengths and weaknesses

Our questionnaire underwent multiple developmental stages and pilot testing for clarity and completeness, incorporating input from pharmacists and researchers across different countries and settings. There was substantial participation and representability achieved by leveraging national and diverse networks across various settings. A limitation is that pharmacists with a positive view on prescribing may have been more inclined to participate. Another limitation is that the participants’ knowledge about existing prescribing models worldwide and a different interpretation of the presented models, tasks, preconditions, benefits and risks might have influenced their responses. Finally, this was a mainly quantitative survey. An in-depth qualitative exploration of participants’ perspectives was not included, although the open text comments were analysed to support quantitative findings.

### Interpretation

The support for pharmacist prescribing in some limited or collaborative model in this study is comparable to previous research findings on the pharmacists’ perspective in other countries [8]. Countries like the UK have successfully implemented pharmacist prescribing, showing positive impact on healthcare efficiency and patient outcomes [11, 31]. Limited or collaborative models may be similar to the prescribing rights that nurses and physician assistants have obtained in the Netherlands [32, 33]. It also appears that participants support the introduction of prescribing authority for themselves more than nurses [34]. In a recently published qualitative study from the Netherlands, citizens who were informed about opportunities for pharmacy prescribing also sketched an ideal future scenario involving a collaborative primary care context where pharmacists could prescribe medication for minor ailments and certain chronic diseases after diagnosis by a general practitioner [35]. There was no major support for independent prescribing as in the UK. Most UK independent pharmacist prescribers work within a collaborative setting (hospitals or general practices) and their prescribing practice is limited to their own clinical competence [36]. As this may not be known to Dutch participants, it could explain their support for a limited or collaborative model. Participants may also primarily see potential for prescribing legislation for tasks that they are already involved in, such as prescription renewals, dosage changes, therapeutic substitution, and therapeutic drug monitoring.

Required preconditions according to pharmacists in this study resemble findings from previous studies. While participants’ express a willingness to take up pharmacist prescribing, patients and healthcare professionals have advocated for a clear separation of responsibilities between physicians and pharmacists [22]. The pharmacist’s professionalism, competence, and trust in collaboration with other healthcare workers is important for successful implementation of pharmacist prescribing [23]. Participants in this study emphasized the importance of having access to health records as a precondition for prescribing, although the survey did not define what access to health records exactly means. Integrated care systems were healthcare professionals share patient data seamlessly, could address concerns about data accessibility. A similar approach as the UK where pharmacists have access to specific sections of electronic health records could be implemented in the Netherlands [8, 37]. Studies from countries like the USA, UK, Canada, and New Zealand show that pharmacist prescribing services are both highly accessible and beneficial in improving access to medicines [12].

Participating pharmacists saw multiple potential benefits and risks of introducing pharmacist prescribing. Other than findings from a previous case study suggested [22], fragmented healthcare was not widely seen as a risk, perhaps due to confidence in shared health records. Pharmacists identified potential benefits such as enhanced professional roles and improved collaboration, which they believe could present specific advantages within the Dutch healthcare context. Most respondents agreed that the conflict of interest when pharmacists assume dual roles as prescribers and dispensers was a risk. Such concerns are often raised in pre-implementation studies, but tend to diminish post-implementation, as participants can reflect on their real-world experiences and the practicalities of pharmacists assuming dual roles [8]. Increased pharmacist workload seemed a primary concern. As the number of pharmacists in the Netherlands is relatively low compared to other European countries [38], this concern might be more relevant to them.

### Implications for policy and research

Pharmacists’ agreement with pharmacist prescribing may be a first step to inform policymakers about the potential introduction of prescribing legislation for pharmacists. Further exploration with other stakeholders is needed to define the most suitable models, possibly focusing on collaborative or independent frameworks. Studies to investigate the patients’ and physicians’ perspectives are currently ongoing. A similar trajectory to that of nurse specialists and physician assistants in the Netherlands could be considered for pharmacist prescribing, starting with a trial phase of existing informal practices as an exception to the law [32]. If the trial phase yields positive results, formal legalization could follow. These findings may provide insights for other countries with similar healthcare systems, though further research is needed to determine the applicability of these results in different international contexts. The results may especially be relevant for regions considering new pharmacist prescribing legislation. Ongoing and planned survey studies in other European countries without formal pharmacist prescribing (e.g., Sweden, Germany, Spain, Slovakia and Turkey), based on the same English version of the questionnaire, may support smoother transitions to pharmacist-led prescribing initiatives across Europe.

## Conclusion

Pharmacists participating in this study are positive towards potential pharmacist prescribing in an independent but limited or collaborative model. These findings support further steps into investigating the potential introduction of pharmacist prescribing in the Netherlands.

## Supplementary Information

Below is the link to the electronic supplementary material.

Supplementary file1 (DOCX 48 KB)
