# Supplementary material for: Pharmacists’ perspectives on potential pharmacist prescribing: a nationwide survey in the Netherlands
Source: Int J Clin Pharm. 2024 Dec 1;47(2):392–402. doi: 10.1007/s11096-024-01842-7 (PMC11920314; doi:10.1007/s11096-024-01842-7)
Supplement: Supplementary file 1 — Supplementary file1 (DOCX 48 KB) [file 11096_2024_1842_MOESM1_ESM.docx]

**Appendix 1**

**Questionnaire 'Pharmacists’ perspectives on pharmacist prescribing' – English translation of the Dutch version that was administered**

The aim of this UPPER study is to gain insight into the perspective of practicing pharmacists in the Netherlands on obtaining prescribing rights. This research is also being conducted in several other European countries, such as Belgium and Sweden.

In some countries, pharmacists have prescribing authority, for example, in the United Kingdom. Pharmacists with prescribing authority are permitted to independently prescribe medications within their own area of expertise (based on competence). In other countries, there are more limited models of prescribing authority for pharmacists. For example, renewal of repeat medication in Denmark and modification of a treatment (e.g., initiating, discontinuing, or adjusting dosage) after diagnosis by a physician in New Zealand. In many other countries, there is no formal prescribing authority for pharmacists.

In this questionnaire, we define practicing pharmacists as pharmacists working in pharmaceutical care for individual patients, such as hospital/outpatient/community pharmacy, or in other healthcare settings. This questionnaire is not intended for non-practicing pharmacists.

In this questionnaire we define pharmacist prescribing as prescribing of medication within the pharmacist’s clinical competence and based on a professional assessment of the patient, including taking responsibility for the prescribed treatment and documenting the process. Having a prescription retrospectively endorsed by a physician is not included here.

This questionnaire contains 15 questions about your opinion on prescribing authority for pharmacists. It takes approximately 15 minutes to complete. The provided answers cannot be traced back to a specific individual and will be treated confidentially. For more information on participating in this research, please refer to the *Participant Information - 'Pharmacist Perspective on Prescribing Authority for Pharmacists' research*.

You may have been invited to participate in this research through various channels (KNMP, NVZA, UPPER). However, it is explicitly intended that you only complete the questionnaire once.

Question 0.

By filling out this survey, I confirm that I am familiar with the provided information and agree that my answers will be used for the intended research.

Question 1. Are you:

- Male
- Female
- Other
- Don't want to answer

Question 2. Year of birth:

Question 3. What pharmacist education(s) have you completed? (You can give multiple answers)

- Master of Pharmacy
- Post-graduate community pharmacy degree and/or registration
- Post-graduate hospital pharmacy degree and/or registration
- Clinical pharmacology specialisation
- Additional community pharmacy specialisation^1^

Other education:

^1^ In the Dutch context, this qualification is referred to as 'kaderapotheker.'

Question 4 - Within which setting are you currently practicing? (You can give multiple answers)

- Community pharmacy (including internet pharmacies)
- Primary care other than community pharmacy (e.g., general practice)
- Secondary care (hospital or specialty care)
- Nursing home

Other:

Additional question, if chosen community pharmacy. The community pharmacy where I work is:

- Owned by a chain
- Independent and part of a franchise/formula
- Independent and not part of a franchise/formula

Other:

Additional question, if chosen hospital pharmacy. The hospital pharmacy where I work is a:

- Academic hospital
- Top clinical hospital
- Generic hospital

Other:

Question 5. What pharmacy function do you currently practice?

- Managing pharmacist
- Second pharmacist
- Hospital pharmacist
- Resident pharmacist
- Non-resident pharmacist

Other:

Question 6 - How often do you have direct patient contact in your current work practice?

- Daily
- Weekly
- Monthly
- (Almost) Never

Question 7 - For how long have you been practicing as a pharmacist? (Enter '0' if you have less than 1 year and '10' if you have more than 10 years of experience)

Question 8. In which province (of The Netherlands) are you a practicing pharmacist?

- Groningen
- Friesland
- Drenthe
- Overijssel
- Gelderland
- Flevoland
- Utrecht
- Noord-Holland
- Zuid-Holland
- Zeeland
- Noord-Brabant
- Limburg

The next questions concern your perspective on different potential models of pharmacist prescribing in the Netherlands. Below you will find **examples** of models that have been implemented in other countries.

| Prescribing based on an **agreement** or **collaboration** | **Independent**, but **limited** prescribing rights | **Independent prescribing** |
| --- | --- | --- |
| 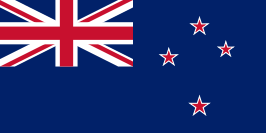  *New-Zealand* | 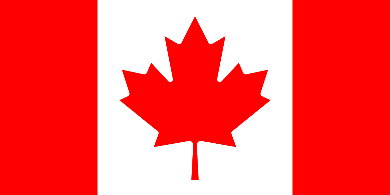  *Ontario, Canada* | 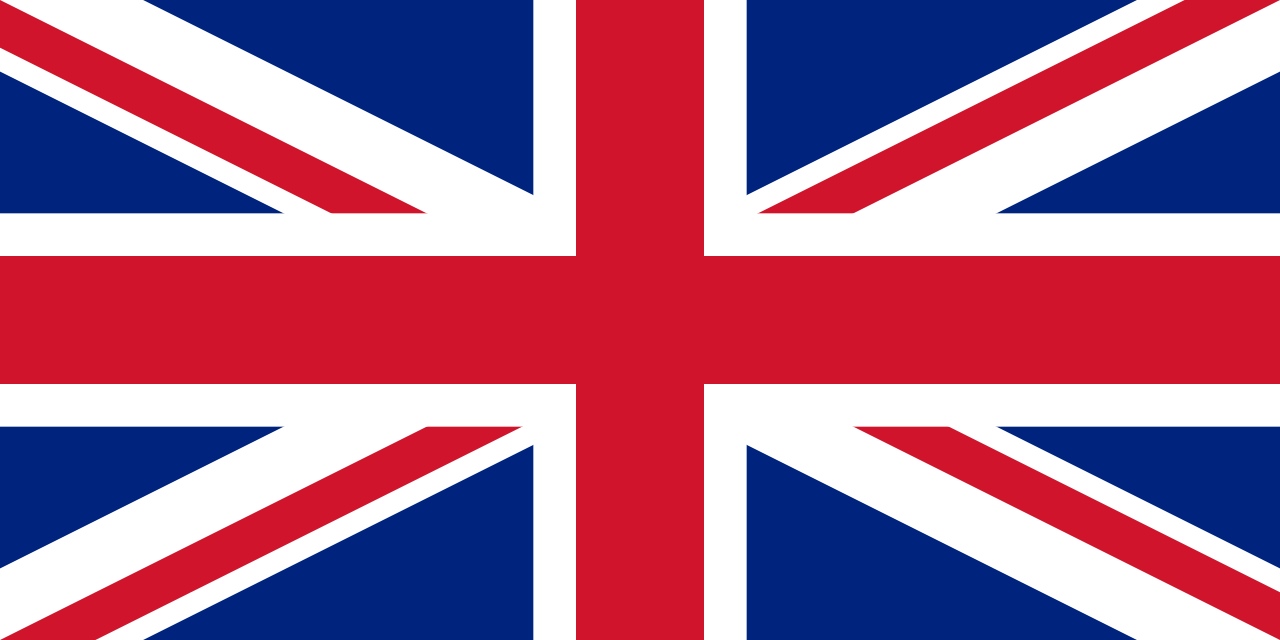 *United Kingdom of Great Britain* |
| - Pharmacists may prescribe in the context of a collaborative health team environment with other healthcare professionals and are not the primary diagnostician | - Pharmacists have the right to prescribe independently (not dependent on an agreement/collaboration with physicians). | - Independent prescribers are responsible and accountable for the assessment of patients with undiagnosed or diagnosed conditions and for decisions about the clinical management, including prescribing. |
| - They can write a prescription for a patient in their care to initiate or modify therapy (including discontinuation or maintenance of therapy originally initiated by another prescriber). | - Pharmacists can renew prescriptions, change dosage (regimen) of an existing prescription, and may prescribe medication to treat a limited number of minor ailments (e.g., allergic rhinitis and urinary tract infections) | - These prescribing rights resemble those that medical doctors have. |
|  |  |  |
| - ***In all prescribing models, pharmacists can only prescribe within the limits of their professional expertise and competence***. | | |

Answer options for questions 9-15

- Disagree
- Somewhat disagree
- Somewhat agree
- Agree
- Don't know / No opinion

Question 9. To what extent do you agree that the following models of prescribing rights for pharmacists should be introduced in the Netherlands?

1. Prescribing based on an **agreement or collaboration** with one or more independent prescribers (e.g., physicians). This agreement could be about prescribing - -for a single patient or certain patients/situations in general.
2. **Independent** (of a physician), but **limited** prescribing rights (e.g., specific patient groups/conditions/drug list/formulary)
3. **Independen**t prescribing rights in patients with **diagnosed conditions** (being responsible and accountable for the assessment of the patient and for decisions about the clinical management, including prescribing)
4. I**ndependent** prescribing rights in both patients with **diagnosed and undiagnosed conditions** (being responsible and accountable for the assessment of the patient and for decisions about the clinical management, including prescribing)

Other model(s) of prescribing rights or comments:

Additional question. If ''Disagree'' is answered on all four alternatives, without adding free text:

What is the most important reason for you not to want to introduce the prescription right for pharmacists in the Netherlands?

- This question then goes directly to question 13 (questions 10 to 11 are skipped)

Question 10. To what extent do you agree that pharmacist prescribing would be suitable in the following settings, if introduced in The Netherlands?

- Community pharmacy
- Primary care other than community pharmacy (e.g., general practice)
- Secondary care (hospital or specialty care)
- Nursing home

Comments:

Question 11. To what extent would you be willing to have the following prescribing rights in the future?

1. Prescribing based on an **agreement or collaboration** with one or more independent prescribers (e.g., physicians). This agreement could be about prescribing for a single patient or certain patients/situations in general.
2. **Independent** (of a physician), but **limited** prescribing rights (e.g., specific patient groups/conditions/drug list/formulary)
3. **Independen**t prescribing rights in patients with **diagnosed conditions** (being responsible and accountable for the assessment of the patient and for decisions about the clinical management, including prescribing)
4. I**ndependent** prescribing rights in both patients with **diagnosed and undiagnosed conditions** (being responsible and accountable for the assessment of the patient and for decisions about the clinical management, including prescribing)

Other model(s) of prescribing rights or comments:

Question 12. If you would get prescribing rights, to what extent would you agree to do the following?

1. **Renew prescriptions** (i.e., renew or repeat an already existing treatment/prescription)
2. **Change the dosage** (form) of an already existing treatment/prescription (e.g., higher/lower dose, other time of the day, or other type of inhaler)
3. **Modify** a treatment for specific **minor ailments** (e.g., hay fever)
4. **Initiate** a treatment for specific **minor ailments** (e.g., hay fever)
5. **Modify** a treatment, after a physician has set a diagnosis for certain **chronic diseases** (e.g., hypertension, diabetes or COPD)
6. **Initiate a treatment**, after a physician has set a diagnosis for certain **chronic diseases** (e.g., hypertension, diabetes or COPD)
7. **Modify** a treatment**,** after a physician has set a diagnosis
8. **Initiate** a treatment, after a physician has set a diagnosis
9. **Initiate** a treatment for a previously **undiagnosed condition** (which you, as a pharmacist, diagnose).

Other options or comments:

Question 13. To what extent do you agree that the following conditions are important for the introduction of pharmacist prescribing in The Netherlands?

1. **Education/Training** for pharmacists (related to prescribing)
2. **Continuing Professional Development** for pharmacists (a formal system or framework that involves the tracking and documenting of skills, knowledge and experience gained, beyond any initial education and training)
3. **Patient support** (e.g., that patient organisations are supportive)
4. **Peer support** (i.e., that the pharmacist professional body is supportive)
5. **Acceptance from physicians**
6. **Acceptance from other healthcare professionals** (e.g., nurses)
7. **Access to health records** (including lab results)
8. **A suitable place for** private patient **consultations**
9. **Interprofessional workplace** (e.g., co-location with physicians)
10. **Enough pharmacy personnel resources**
11. **Funding/Remuneration** for the time the pharmacist spends on prescribing
12. **Regulation/Legislation** that enables pharmacist prescribing

Other condition(s) or comments:

Question 14. To what extent do you agree that the following benefits would be the result of pharmacist prescribing?

1. **Increased accessibility** to medication treatment for patients
2. **Improved health outcomes** for patients
3. **Improved patient safety**
4. **Reduced prescribing errors**
5. **Decreased healthcare costs** (for patients and/or society)
6. **Reduced workload for other prescribers** (other than pharmacists)
7. **Increased collaboration with other healthcare professionals**
8. **Enhanced professional position** of pharmacists in healthcare
9. **Increased pharmacy income**
10. **Increased work satisfaction** for pharmacists

Other benefits or comments:

Question 15. To what extent do you agree that the following risks would be the result of pharmacist prescribing?

1. **Conflict of interest** with pharmacists acting both as prescribers and dispensers
2. **Decreased patient health outcomes**
3. **Decreased patient safety**
4. **Increased prescribing errors**
5. **Increased healthcare costs** (for patients and/or society)
6. **Increased workload** **for pharmacists**
7. **Decreased collaboration with other healthcare** **professionals**
8. **Fragmented** **healthcare**
9. **Decreased pharmacy income**
10. **Increase in administrative burden for** **pharmacists**

Other risks or comments:

Additional question. Please write any other comments you have on pharmacist prescribing and relating issues:

**Appendix 2 – Subgroup analyses**

**Subgroup analysis by setting**

Subgroup analysis based on setting (Table S1) includes questions about agreement on the introduction of various models in the Netherlands (Table S2).

*Table S1: Setting where the pharmacist is working (N=625)*

| **Setting pharmacist** | **n (percentage)** |
| --- | --- |
| Community pharmacy (including internet pharmacy) | 432 (69.1%) |
| Outpatient hospital pharmacy | 24 (3.8%) |
| Hospital pharmacy | 125 (20.0%) |
| Other^1^ | 44 (7.0%) |

*^1^Institution (e.g., nursing home), general practice, or combinations of the settings above.*

*Table S2: Subgroup analysis on questions regarding the setting and agreement on the introduction of various models in the Netherlands*

|  | **A) Prescribing based on an agreement or collaboration** | **B) Independent, but limited** | **C) Independent for diagnosed conditions** | **D) Independent for diagnosed and undiagnosed conditions** |
| --- | --- | --- | --- | --- |
| **All** | 474/625=75.8% | 538/625=86.1% | 470/625 =75.2% | 245/625=39.2% |
| **Community pharmacy** | 332/433=76.7% | 395/433=91.2%^2^ | 368/433=85.0%^2^ | 209/433=48.3%^2^ |
| **Outpatient hospital pharmacy** | 19/24= 79.2% | 21/24=87.5% | 15/24=62.5% | 3/24=12.5% |
| **Inpatient hospital pharmacy** | 87/126= 69.0% | 86/126=68.3%^2^ | 61/126=48.4%^2^ | 25/126=19.8%^2^ |
| **Other** | 33/42 = 78.5% | 36/42 = 85.7% | 30/42= 71.4% | 8/42=19.0%^2^ |
| **Chi-square p-value^1^** | p= 0.540 | p<0.001 | p<0.001 | p<0.001 |

*1: p-value adjusted to account for multiple comparison
2: Significant difference (p<0.05) between expected and observed in this subgroup according to post hoc test after adjustment for multiple comparisons.*

**Subgroup analysis by direct patient contact**

Subgroup analysis based on personal contact with patients (Table S3) and questions about agreement on the introduction of various models in the Netherlands (Table S4).

*Table S3: Direct patient contact (N=625)*

| **Frequency of direct patient contact** | **n (percentage)** |
| --- | --- |
| Daily | 421 (67.4%) |
| Weekly | 73 (11.7%) |
| Monthly | 37 (5.9%) |
| (Almost) never | 94 (5.9%) |

*Table S4: Subgroup analysis on questions regarding personal contact with patients and agreement on the introduction of various models in the Netherland*

|  | **A) Prescribing based on an agreement or collaboration** | **B) Independent, but limited** | **C) Independent for diagnosed conditions** | **D) Independent for diagnosed and undiagnosed conditions** |
| --- | --- | --- | --- | --- |
| **All** | 474/625= 75.8% | 538/625= 86.1% | 470/625= 77.4% | 245/625= 39.2% |
| **Daily** | 320/421= 76.0% | 380/421= 90.3%^2^ | 354/421=84.1%^2^ | 200/421=47.5%^2^ |
| **Weekly** | 61/73= 83.6% | 66 /73= 90.4% | 52/73= 71.2% | 19/73= 26.0% |
| **Monthly** | 26/37= 70.3% | 29/37= 78.4% | 20/37= 54.1%^2^ | 28/37= 75.7% |
| **(Almost) never** | 64/94= 68.1% | 63/94= 67.0%^2^ | 48/94= 51.1%^2^ | 17/94= 18.1%^2^ |
| **Chi square p-value^1^** | p=0.309 | p<0.001 | p<0.001 | p<0.001 |

*1: p-value adjusted to account for multiple comparison
2: Significant difference (p<0.05) between expected and observed in this subgroup according to post hoc test after adjustment for multiple comparisons.*

**Subgroup analysis by years of working experience**

Subgroup analysis based on years of working experience (Table S5) and questions about agreement on the introduction of various models in the Netherlands (Table S6).

*Table S5: Frequency of working experience (N=625)*

| **Frequency of years of working experience** | n (percentage) |
| --- | --- |
| 0-5 years | 107 (17.2%) |
| 6-9 years | 95 (15.2%) |
| >10 years | 423 (67.7%) |

*Table S6: Subgroup analysis on questions regarding years of working experience with patients and agreement on the introduction of various models in the Netherlands.*

|  | **A) Prescribing based on an agreement or collaboration** | **B) Independent, but limited** | **C) Independent for diagnosed conditions** | **D) Independent for diagnosed and undiagnosed conditions** |
| --- | --- | --- | --- | --- |
| **All** | 474/625= 75,8% | 538/625 = 86.1% | 470/625 = 75.2% | 245/625 = 39.1% |
| **0-5 years** | 89/107= 83.2% | 96/107 = 89.7% | 79/107 = 73.8% | 34/107 = 31.8% |
| **6-9 years** | 76/95 = 80.0% | 88/95 = 92.6% | 79/95 = 83.2% | 32/95 = 33.7% |
| **>10 years** | 306/423= 72.3% | 354/423 = 83.7% | 316/423 = 74.7% | 179/423 = 42.3% |
| **Chi square p-value^1^** | p=1.120 | p=0.077 | p=0.587 | p=0.389 |

*1: p-value adjusted to account for multiple comparison*
